# Knowledge, practices, and concerns of tuberculosis healthcare workers at primary settings in western China: a multi-center cross-sectional study

**DOI:** 10.1186/s40249-025-01390-w

**Published:** 2025-12-04

**Authors:** Jiani Zhou, Jian Wang, Qingning Huang, Long Luo, Wei Chen, Qingya Wang, Geng Wang, Shili Liu, Xi Chen, Quan Yuan, Haonan Bai, Ying Li

**Affiliations:** 1 Department of Social Medicine and Health Service Management, Army Medical University (Third Military Medical University), Shapingba District, Chongqing Municipality, 400038 China; 2 Center for Disease Control and Prevention of Xizang, Chengguan District, Lhasa, 850000 Xizang China; 3 Institute for Tuberculosis Prevention and Control, Guizhou Center for Disease Control and Prevention, Guiyang, 550004 Guizhou China; 4 Department of Districts and Counties, Chongqing Institute of TB Prevention and Treatment, Jiulongpo District, Chongqing, 400050 China

**Keywords:** Tuberculosis, Primary healthcare, Healthcare worker, Knowledge, Practice, China

## Abstract

**Background:**

Tuberculosis (TB) remains a major global health challenge, and China bearing the world’s third-highest burden. TB healthcare workers (TB-HCWs) in primary healthcare (PHC) settings are pivotal for implementing the national TB Control Program (TCP). This study aimed to develop a TB knowledge assessment questionnaire and systematically evaluate TB-HCWs’ knowledge, practices, and perceived concerns regarding TCP implementation in western China.

**Methods:**

A 30-item TB knowledge questionnaire was developed through item generation, expert consultation, and pilot testing. A multicenter cross-sectional study was conducted among TB-HCWs in Chongqing Municipality, Guizhou Province, and Xizang Zizhiqu from February 2022 to July 2023, using multistage stratified random sampling. A structured survey assessed demographics, TB knowledge, TCP practices, and perceived concerns. Multilevel logistic regression identified factors associated with TCP implementation.

**Results:**

Among 2807 TB-HCWs, overall TB knowledge was low (38.4%), particularly for TB Treatment (25.1%). Rural TB-HCWs performed better in case management (41.3% vs. 40.1%; *P* < 0.05) and health education (50.0% vs. 47.1%; *P* < 0.001), while urban TB-HCWs scored higher in case detection and TB treatment (42.3% vs. 40.9%; *P* < 0.05). TCP practice implementation generally fell short of national standards, though urban HCWs achieved required levels in first-home visiting (≥ 90%) and health education (≥ 85%). Workforce-intensive services, particularly directly observed therapy, were suboptimally delivered in both settings, especially rural (< 70%). Positive working attitudes and working satisfaction predicted higher implementation across all dimensions [odds ratio (*OR*) > 1], while rural settings and infrequent training (≤ 1/half-year) were negative predictors (*OR* < 1). Key concerns included inadequate training, poor public/patient cooperation, insufficient workforce, weak coordination with TB-designated hospitals, and lack of incentives.

**Conclusions:**

TB-HCWs in western China face substantial gaps in knowledge and practice that hinder effective PHC-based TCP delivery. Targeted and frequent training, context-specific and patient-centered adherence strategies, improved institutional support, and strengthened community engagement are needed. Future longitudinal studies should evaluate the effectiveness and long-term impact of these interventions to accelerate progress toward national and global End TB targets.

**Graphical Abstract:**

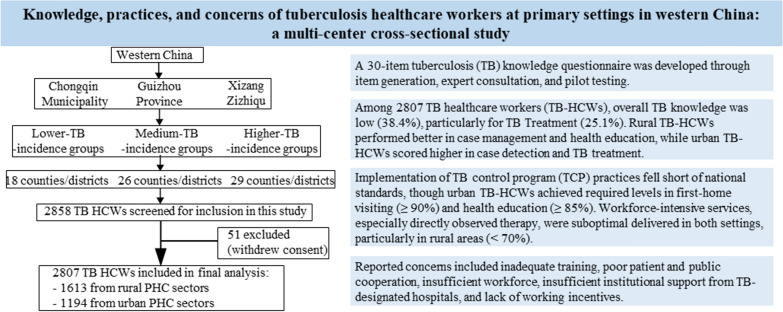

**Supplementary Information:**

The online version contains supplementary material available at 10.1186/s40249-025-01390-w.

## Background

Tuberculosis (TB) remains a major global health challenge, with an estimated 10.8 million incident cases and 1.3 million deaths reported globally in 2023, making it the leading infectious cause of mortality, surpassing both COVID-19 and HIV/AIDS [1]. The global TB incidence rate was 134 per 100,000 population, indicating only an 8.3% reduction since 2015—far below the 50% reduction targeted set by the End TB Strategy for 2025 [1, 2]. Among incident cases, 6.1% were co-infected with HIV and 3.7% had multidrug-resistant or rifampicin-resistant TB (MDR/RR-TB) [1]. China continues to face enormous challenges due to its large population. Although its national incidence rate (52 per 100,000) is less than half the global average, China reported 741,000 new cases in 2023, accounting for 6.8% of global cases and ranking third worldwide [1]. TB burden is disproportionately higher in western China compared with central and eastern regions, and approximately 10% of counties or districts report incidence rates exceeding 100 per 100,000 [3].

A major obstacle to end TB is the prolonged treatment duration, 6–9 months of daily medication for drug-susceptible TB (DS-TB) and up to 20 months for MDR/RR-TB, which makes adherence critical yet vulnerable. Improving adherence has been a primary focus of the World Health Organization (WHO), which recommends patient-centered and community-based strategies such as directly observed therapy (DOT) to improve treatment compliance and success [2, 4].

In line with WHO recommendations, China has demonstrated a strong commitment to end TB. Since 2012, it has implemented a nationwide integrated TB control model based on tripartite collaboration between Centers for Disease Control (CDCs), TB-designated hospitals, and primary healthcare (PHC) sectors [5, 6]. Under this model, CDCs oversee program governance, surveillance, technical training, and health promotion; TB-designated hospitals provide clinical diagnosis and treatment; and PHC sectors conduct community-based screening and referral of TB patients and persons with presumed TB, case tracing, case management, and health education [7].

PHC sectors in China are community-based institutions—comprising community health centers and community health service stations in urban areas, and township hospitals and village clinics in rural areas—which play an essential role in TB control [7–11]. They deliver key TB Control Program (TCP) services, including active case detection and referral, contact tracking, case management, and TB health education [12–14]. In this study, healthcare workers (HCWs) in PHC sectors, responsible for TCP implementation, are referred to as TB-HCWs.

TCP services delivered by TB-HCWs in PHC sectors (PHC-based TCP) are crucial for reducing community transmission, promoting early detection and treatment, ensuring adherence to standardized therapy, preventing drug-resistant TB (DR-TB), and reducing catastrophic health expenditures. Since 2015, PHC-based TCP services have been formally incorporated into China’s Basic Public Health Service (BPHS) package, a national initiative to ensuring equitable access to essential public services [15, 16]. The implementation of PHC-based TCP has led to substantial improvements in TB control, including higher case detection and case management rates, better treatment outcomes, reduced incidence, and improved public awareness in several high-burden regions in China and internationally [17–27].

However, previous studies have reported that PHC-based TCP faced multiple challenges, including insufficient facilities, shortages of healthcare providers, heavy workloads, inadequate TB care competencies, limited training, insufficient work incentives, lack of patient- or people-centered care, weak multisector coordination, social discrimination, and low public awareness of TB [8, 9, 18, 28–33]. These barriers contribute to delayed case detection, increased transmission risks, poor adherence, and unfavored treatment outcomes. In western China, more than 50% of TB patients remained undetected, and nearly two-thirds of close contacts were not screened [8, 33]. Only 17% of DS-TB cases and 39.6% of DR-TB cases received standardized case management, and alarmingly, about 40% of patients declined case management altogether, with some refusing TCP services entirely [32].

The TB care competency of TB-HCWs represents an integrated combination of knowledge, skills, and attitudes required for effective delivery of PHC-based TCP services. Existing research on PHC-based TCP knowledge and skills among TB-HCWs remains limited. To date, only five studies from China—conducted in Hefei, Xinjiang, Xizang, and Shanghai—have reported outcomes of TB-related core knowledge among TB-HCWs, focusing mainly on contagiousness, suspected symptoms, case referral, policy awareness, and curability [34–38]. The Hefei study reported an 80% awareness rate of three core TB knowledge items among TB-HCWs, with high staff turnover associated with lower awareness [34]. In Jiading District of Shanghai, the general awareness rate of core TB knowledge was 76.5% [35]. Another study across six districts in Shanghai reported a 60.7% awareness rate of general TB knowledge among PHC-based TB-HCWs [36]. In Naqu County, Xizang, more than 90% of TB-HCWs demonstrated inadequate TB knowledge [37]. In Xinjiang, the awareness rate of TB knowledge was 74.2% among TB-related healthcare professionals, including TB-HCWs [38]. Notably, among these five studies, only one explicitly reported using structured assessment methods, such as single-choice or fill-in-the–blank questions, to measure TB-HCWs’ TB knowledge [38]. Most studies assessed general TB knowledge rather than knowledge specifically related to PHC-based TCP delivery.

Core competencies in TB control—particularly patient-centered communication, psychosocial support, and TB health education delivery—are critical determinants of TCP quality and effectiveness. Evidence indicates that mastery of these competencies directly influences patient trust in TB-HCW and acceptance of services [9, 30, 39]. To the best of our knowledge, no study has systematically assessed the knowledge, practices, and attitudes of TB-HCWs regarding PHC-based TCP, and structured instruments to comprehensively evaluate TB-HCWs’ TCP competency are lacking.

This study therefore had two objectives. The one was to develop a structured assessment instrument to measure TB knowledge among TB-HCWs, providing an adaptable instrument for evaluating TB care competency in PHC settings; the other one was to assess the knowledge, practices, and perceived concerns of TB-HCWs regarding PHC-based TCP implementation, thereby generating evidence to inform interventions aimed at improving the quality of TCP services.

## Methods

### Overall study design

This multicenter cross-sectional study was conducted between February 2022 and July 2023 across three provincial-level administrative divisions (PLADs) in western China—Chongqing Municipality (Chongqing), Guizhou Province (Guizhou), and Xizang Zizhiqu (Xizang)—where the prevalence of TB exceeds the national average. The study design and reporting followed the Strengthening the Reporting of Observational Studies in Epidemiology (STROBE) guidelines for cross-sectional research (Supplementary Table S1) [40].

### Study setting, sampling, and sample size

**Fig. 1:**
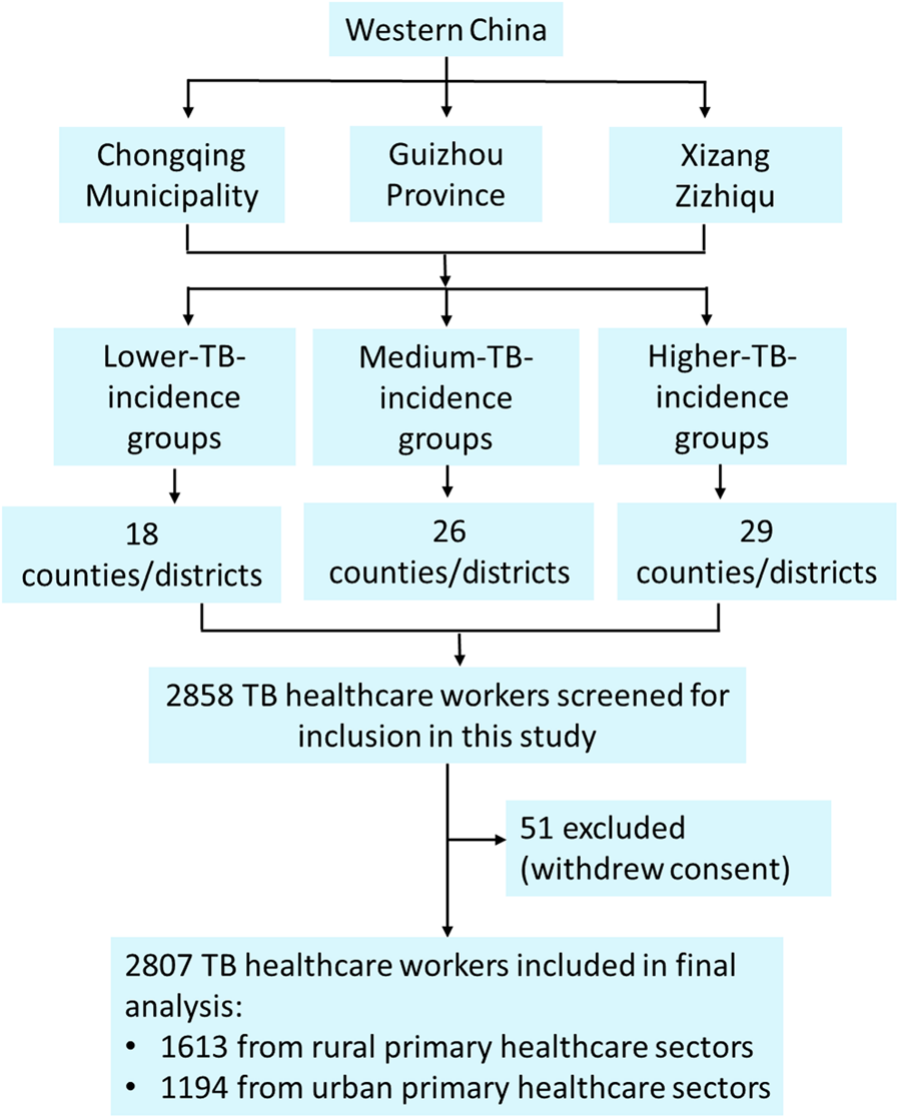
The study flowchart. *TB* tuberculosis.

A multistage stratified random sampling method was employed to select PHC sectors in the three selected PLADs (Fig. 1). Prevalence of TB in western China is significantly higher than that in the central and eastern regions [41, 42]. These PLADs were purposively selected to represent different levels of socioeconomic development and TB burden. Chongqing represents regions with stronger socioeconomic status and a lower TB burden; Guizhou represents regions with moderate socioeconomic development and a medium TB burden; and Xizang represents areas with lower socioeconomic development and the highest TB burden [42–45]. Chongqing reported a TB incidence of 61.7 per 100,000 in 2021, above the national average rate of 45.4 per 100,000 [46]. Guizhou and Xizang reported higher burdens, at 102.5 per 100,000 in 2019 and an average of 147 per 100,000 during 2010–2019, respectively [47, 48].

Within each PLAD, all counties or districts were stratified into three categories of TB incidence (lower, medium, and higher) based on thresholds defined by provincial surveillance data. In Chongqing, incidence thresholds were < 30, 30–50, and > 50 per 100,000 for urban areas, and < 30, 30–70, and > 70 per 100,000 for rural areas [49]. In Guizhou, thresholds were < 55, 55–100, and > 100 per 100,000, whereas in Xizang they were < 55, 55–150, and > 150 per 100,000 [50]. From each incidence stratum, counties or districts were randomly selected using a proportional allocation approach, ensuring that the number of selected units reflected distribution of PHC sectors across stratum. In total, 73 counties or districts were included and subsequently categorized as either urban or rural.

Sample size was estimated using the stratified sampling formula [51, 52]:

$$n=\frac{\sum {W}_{i}^{2}{S}_{i}^{2}/{w}_{i}}{V+\sum {W}_{i}{S}_{i}^{2}/N}$$

where *n* is the minimum total sample size; *W*_*i*_ is the weight of the population in stratum *i;* ${S}_{i}^{2}$ is the variance within stratum *i*; ${w}_{i}$ represents the allocated sample size for stratum *i*; *V* is the desired variance of the estimator; *N* is the total population size. According to the number of PHC sectors in Chongqing, Guizhou, and Xizang, and assuming at least one TB-HCWs per PHC sector, *N* was set to 60,000 [53–55]. We assigned equal weights (*W*_*1*_ = *W*_*2*_ = *W*_*3*_ = 1/3) and set $S$_1_ = 70, $S$_2_ = 65, and $S$_3_ = 60, reflecting the distribution of TCP delivery across the three incidence-burden categories. The variance component was defined as *V* = (δ/u_α/2_)^2^, where δ is the error tolerance and u_α/2_ is the critical value from the standard normal distribution. We set δ = 3.5 and u_α/2_ = 1.96 (corresponding to a 5% significance level). The calculated minimum sample size was approximately 1300, which was rounded up to 1500. Accordingly, the minimum sample size for each stratum was 500. Ultimately, among 2858 eligible TB-HCWs recruited, 2807 completed the survey, yielding a response rate of 98.2%.

### Study participants and recruitment

Eligible participants were TB-HCWs engaged in delivering TCP services at PHC sectors within the selected counties or districts. In China, PHC sectors refer to community-based institutions, including community health centers and community health service stations in urban areas, and township hospitals and village clinics in rural areas. These facilities play a vital role in TB control.

Inclusion criteria were: (1) employed in a PHC sectors located within a study site; (2) responsibility for any aspect of PHC-based TCP delivery; and (3) provision of informed online consent and independently completion of the online survey. Exclusion criteria were: (1) HCWs not directly involved in PHC-based TCP activities (e.g., administrative or non-TB staff); (2) temporary or trainee staff without independent TCP responsibilities; and (3) incomplete or duplicate questionnaires, which were removed during data quality checks.

Recruitment was facilitated by local CDCs and TB dispensaries, which distributed the survey link and quick response code to leaders of PHC sectors in the study sites. PHC leaders then shared this information with frontline TB-HCWs. The role of CDCs, TB dispensaries, and PHC leaders was limited to survey dissemination. They had no access to individual responses or the final dataset.

Participation was voluntary and anonymous. All data collection, storage, and analysis were conducted independently by the research team as a third party. The first page of the online questionnaire provided a study overview and informed consent form. Participants were required to select “Yes, I consent” before proceeding. Eligibility was verified through screening questions (e.g. job position, TCP duties), and ineligible responses were automatically excluded. Completed questionnaires were reviewed by the research team for quality control, including duplicate detection, response-time checks, and reliability assessment.

## Data collection

### TB knowledge assessment questionnaire development

The TB knowledge assessment questionnaire was developed through a structured, multi-stage process. Stage 1: An initial pool of 20 items was drafted based on China’s *National TB Prevention and Control Guidance* and related technical specifications [7, 15]. These items covered three domains: (1) Case Detection and Referral, (2) Anti-TB Treatment, and (3) Case Management. Two investigators (JZ and LY) independently reviewed and mapped the items to ensure their alignment with the core competencies required for PHC-based TCP.

Stage 2: The item pool was refined through iterative consultations with multidisciplinary stakeholders, including TB experts from CDC, TB-HCWs from TB dispensary, frontline TB-HCWs, and PHC administrators. Four new items were added, covering (1) reimbursement policies for DR-TB, (2) hepatotoxicity symptoms as an adverse drug reaction, (3) case management for smear-negative TB patients, and (4) criteria for school suspension in student TB cases. A new domain—TB Health Education—was also introduced, adding six additional items, for a total of 30 items.

Stage 3: Items were designed as a multiple-choice, true-false, or single-choice question to assess understanding rather than recall of trivial details. Two scoring metrics were defined: (1) item-specific correct rate (correct responses per item/total responses per item), and (2) overall correct rate (total correct responses/total possible responses).

Stage 4: A pilot test was conducted among 56 TB-HCWs in Pengshui County, Chongqing. The questionnaire demonstrated good internal consistency (Cronbach’s α = 0.870 > 0.80), confirming reliability.

The final 30-items TB knowledge assessment questionnaire covered four domains: Case Detection and Referral (8 items), TB Treatment (8 items), Case Management (8 items), and TB Health Education (6 items). The item set comprised 4 single-choice, 5 true-false, and 21 multiple-choice questions (Table 1). Each correct response was awarded one point.

**Table 1:** The TB knowledge assessment questionnaire for TB healthcare workers in primary health care sectors

Themes	Knowledge items	Question type	Point
1. Case detection and referral	1. Main symptoms of suspected TB cases	Multiple-choice	1
	2. Ohter common symptoms of suspected TB cases	Multiple-choice	1
	3. Mehtods of detecting suspected TB cases	Multiple-choice	1
	4. Screening scope of key populations for TB	Multiple-choice	1
	5. Screening scope of suspected DR-TB cases	Multiple-choice	1
	6. Referral of suspected TB cases	Multiple-choice	1
	7. Medical insurance reimbursement policy for suspected TB cases	Multiple-choice	1
	8. Scope of close contacts of TB cases	Multiple-choice	1
2. TB treatment	9. Eligibility for free TB treatment	Multiple-choice	1
	10. Reimbursement policy for DS-TB	Multiple-choice	1
	11. Reimbursement policy for DR-TB	Single-choice	1
	12. Gold standard for TB diagnosis	Single-choice	1
	13. Commonly used first-line anti-TB drugs	Multiple-choice	1
	14. Common side-effects of anti-TB drugs	Multiple-choice	1
	15. Symptoms of hepatotoxicity adverse reactions	Multiple-choice	1
	16. Principles of TB treatment	Multiple-choice	1
3. Case management	17. Eligible patients of case management	Multiple-choice	1
	18. Case management methods for newly diagnosed and smear-positive cases	Single-choice	1
	19. Case management methods for newly diagnosed and smear-negative cases	Single-choice	1
	20. Main supervisors of case management	Single-choice	1
	21. Supervision contents	Single-choice	1
	22. Time for sputum re-examination of newly treated cases	Multiple-choice	1
	23. Time for sputum examination and re-examination of re-treated cases	Multiple-choice	1
	24. Criteria for TB student patients to suspend school	Multiple-choice	1
4. TB health education	25. Disinfection manners of *Mycobacterium* *tuberculosis*	Multiple-choice	1
	26. Transmission route of TB	True-false	1
	27. Manners of preventing the spread of TB	True-false	1
	28. TB cure rate	True-false	1
	29. Criteria for judging the infectivity of TB	True-false	1
	30. Identifying side-effects of anti-TB drugs	True-false	1
Total points			30

*TB* tuberculosis, *DR-TB* drug-resistant tuberculosis, *DS-TB* drug-susceptible tuberculosis

### Main survey development

A structured online survey was developed in Chinese, comprising four sections. First, demographic and occupational information was collected based on previous studies [8, 21], including gender, age, education level, professional title, years of PHC experience, number of BPHS tasks, working satisfaction, working attitude, and frequency of TCP training. Work satisfaction was assessed using the question: “How satisfied are you with your current work position?” with a five-point Likert scale (very satisfied, satisfied, neutral, dissatisfied, and very dissatisfied). Work attitude was assessed with the question: “Do you intend to continue working in your current position in the future?” using a five-point Likert scale (very likely, likely, unsure, unlikely, very unlikely).

Second, TB knowledge was assessed utilizing the developed TB knowledge assessment questionnaire. Third, TCP practices were evaluated using a 23-item questionnaire developed from *China’s National TB Prevention and Control Guidance* and BPHS standards [7, 15]. The questionnaire covered four domains: (A) Case Detection, Referral, and Tracking (7 items), (B) First-home Visiting (4 items), (C) Case Management (10 items), and (D) Health Education (2 items). For each item, the implementation rate was calculated as the proportion of respondents reporting service delivery (‘delivered’/total valid responses).

Finally, participants’ perceived barriers and challenges in TCP implementation were assessed to capture the perspective of frontline TB-HCWs. Pilot testing was conducted among 261 TB-HCWs from PHC sectors, confirming good reliability and validity. Internal consistency, assessed via Cronbach’s α (range: 0–1; > 0.8 indicating high reliability), demonstrated excellent reliability (α = 0.910). Structural validity, evaluated using item-level (I-CVI = 0.850–1.000) and questionnaire-level content validity indices (S-CVI = 0.960), confirmed robust questionnaire design.

### Data analysis

Data cleaning was conducted in Microsoft Excel (Microsoft Corp., Redmond, WA, USA). Statistical analysis were performed using SPSS software version 26.0 (IBM Corp., Armonk, NY, USA) and GraphPad Prism version 8.0 (GraphPad Software, San Diego, CA, USA). Missing data were excluded from the analysis. Descriptive statistics were used to summarize participant characteristics, knowledge, practices, and concerns in TCP delivery. Urban-rural comparisons were performed using Pearson’s Chi-square test or Fisher’s exact test, as appropriate.

To identified factors associated with TCP implementation, four TCP dimensions were evaluated: (A) Case Detection, Referral, and Tracking, (B) First-home Visiting, (C) Case Management, and (D) Health Education. Binary variables were created following Chinese national TCP standards [3]: Dimension A was coded 1 if the implementation rate ≥ 80% and 0 otherwise; Dimension B was coded 1 if the implementation rate ≥ 90% and 0 otherwise; Dimension C was coded 1 if the implementation rate ≥ 90% and 0 otherwise; Dimension D was coded 1 if the implementation rate ≥ 85% and 0 otherwise. Working satisfaction was coded 1 for ‘satisfied’ (very satisfied/satisfied) and 0 for ‘dissatisfied’ (neutral/dissatisfied/very dissatisfied). Working attitude was coded 1 for ‘positive’ (very likely/likely to continue) and 0 for ‘negative’ (unsure/unlikely/very unlikely to continue). Multivariable logistic regression was performed to identify factors associated with TCP implementation, incorporating both TB-HCW and PHC-level variables. Results were presented as forest plots. Statistical significance was set at *P* < 0.05 (two-tailed).

### Definition

TB is caused by the bacillus *Mycobacterium tuberculosis*, which spreads when individuals with active TB release bacteria into the air, for example, by coughing [1]. The WHO recommends a six-month treatment regimen for DS-TB, with an expected treatment success rate of 88% [1]. DR-TB is classified into five categories: isoniazid-resistant TB, RR-TB, MDR-TB, pre-extensively drug-resistant TB (pre-XDR-TB), and XDR-TB [1]. Rifampicin is the most effective first-line anti-TB drug. RR-TB refers to TB resistant to rifampicin; MDR-TB is resistant to rifampicin and isoniazid; pre-XDR-TB is resistant to rifampicin and any fluoroquinolone (a class of second-line anti-TB drug); and XDR-TB is resistant to rifampicin, any fluoroquinolone, and at least one of either bedaquiline or linezolid [1]. The WHO recommends treatment regimens for MDR/RR-TB lasting 9–20 months, depending on the resistance profile, with a reported treatment success rate of 68% among initiating therapy in 2021 [1]. TB-HCWs refer community health workers and village doctors who provide community-based TB care, including active screening, referral of presumptive TB cases, case tracing, case management, and TB health education for patients and residents [7].

### Ethical considerations

The study was conducted according to the principles in the Declaration of Helsinki. The project proposal was approved by the Institutional Review Board of Army Medical University (Third Military Medical University), Chongqing, China (Grant No. 2021-03-02 and No. 2024-37-02). Participation in the study was voluntary, and informed consent was obtained from all participants.

## Results

### Participant characteristics

A total of 2807 TB-HCWs were included in the final analysis, with 57.5% from rural and 42.5% from urban PHC sectors (Table 2). Clear rural-urban disparities were observed. Rural TB-HCWs were predominantly female (56.6% vs. 44.1%), had longer PHC experience (≥ 5 years: 59.8% vs. 45.1%), and undertook a heavier BPHS workload (≥ 5 tasks: 70.2% vs. 29.6%). In contrast, urban TB-HCWs were younger (aged 20–29: 42.2% vs. 16.3%), better educated (junior medical college or above: 73.4% vs. 47.6%), had higher professional titles (60.0% vs. 36.3%), and participated more frequently in training (≥ 3 times per half-year: 58.7% vs. 35.3%). Regarding work attitudes, rural TB-HCWs reported greater willingness to continue current position (74.5% vs. 65.7%), whereas urban TB-HCWs expressed higher work satisfaction (55.0% vs. 49.5%). Detailed regional characteristics are presented in Supplementary Table S2.

**Table 2:** Sociodemographic characteristics of the study participants (*n* = 2807), *n* (%)

Characteristics	Primary healthcare sector levels		*χ*² value	*P* value
	Rural (*n *= 1613)	Urban (*n *= 1194)		
Gender			42.673	< 0.001
Female	913(56.6)	527(44.1)		
Male	700(43.4)	667(55.9)		
Age, years			376.755	< 0.001
20–29	263(16.3)	504(42.2)		
30–39	337(20.9)	359(30.1)		
40–49	638(39.6)	242(20.3)		
> 49	375(23.2)	89(7.5)		
Education level			535.749	< 0.001
Technical secondary school/below	845(52.4)	318(26.6)		
Junior medical college	589(36.5)	544(45.6)		
Undergraduate college/above	179(11.1)	332(27.8)		
Working title			142.859	< 0.001
None	1027(63.7)	490(41.0)		
Junior	474(29.4)	551(46.1)		
Intermediate/above	112(6.9)	153(12.8)		
Number of BPHS undertake			470.159	< 0.001
≤ 2	312(19.3)	442(37.0)		
3–4	169(10.5)	398(33.3)		
≥ 5	1132(70.2)	354(29.6)		
Working years			59.749	< 0.001
≤ 2	433(26.8)	422(35.3)		
3–4	216(13.4)	233(19.5)		
≥ 5	964(59.8)	539(45.1)		
Working satisfaction			8.284	0.004
Satisfied	799(49.5)	657(55.0)		
Dissatisfied	814(50.5)	537(45.0)		
Working attitude			26.015	< 0.001
Positive	1202(74.5)	784(65.7)		
Negative	411(25.5)	410(34.3)		
TCP training frequency			151.084	< 0.001
≤ 1/half-year	279(17.3)	319(26.7)		
2/half-year	387(24.0)	454(38.0)		
≥ 3/half-year	947(58.7)	421(35.3)		
TB burden group			41.722	< 0.001
Lower	513(31.8)	516(43.2)		
Medium	570(35.3)	321(26.9)		
Higher	530(32.9)	357(29.9)		

*Note* *BPHS* basic public health service, *TCP* tuberculosis control program, *TB* tuberculosis. Analysis through Pearson Chi-square (*χ*²) test. Percentages may not total 100 owing to rounding.

### TCP knowledge of TB-HCWs

**Fig. 2:**
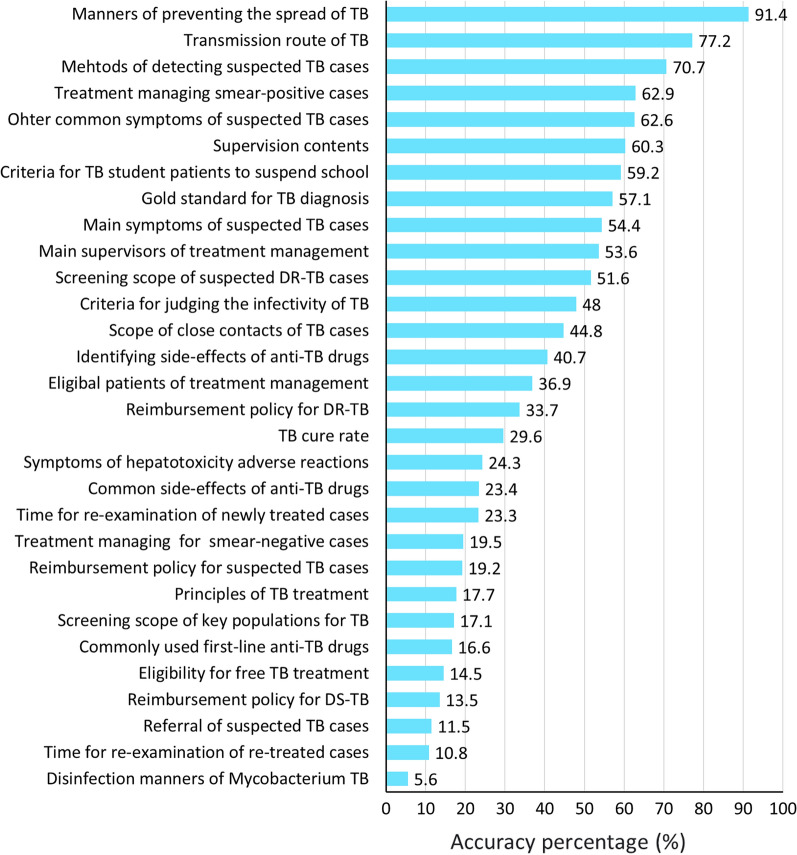
Knowledge associated with TB control program delivery by the study participants, (*n* = 2807). Percentages may not total 100 owing to rounding. *TB* tuberculosis, *DS-TB* drug-susceptible TB, *DR-TB* drug-resistant TB

A 30-items TB knowledge assessment questionnaire among TB-HCWs was developed, covering four domains, with a good internal consistency (Cronbach’s α = 0.870 > 0.80) and good reliability. Overall, TB knowledge among TB-HCWs was low, with a mean correct response rate of 38.4% (Table 3). Urban TB-HCWs scored slightly higher than rural counterparts (38.7% vs. 37.3%; *P* < 0.05). Across domains, knowledge was highest in Health Education (48.7%), followed by Case Detection and Referral (41.5%) and Case Management (40.8%). Knowledge of TB treatment was lowest, with only 25.1% of correct responses. Ten items demonstrated critical knowledge gaps (< 20% correct) (Fig. 2), notably regarding disinfection manners for *Mycobacterium tuberculosis* (5.6%), re-examination schedules for re-treated cases (10.8%), referral of suspected cases (11.5%), and reimbursement policies for DS-TB (13.5%). Most deficiencies were concentrated in the TB Treatment domain.

**Table 3:** TB knowledge of participated TB healthcare workers, *n* (%)

Themes	Knowledge items	Primary healthcare sector levels		χ² value	*P *value
		Rural (*n *= 1613)	Urban (*n *= 1194)		
A. Case detection and referral	1. Main symptoms of suspected TB cases	930(57.7)	596(49.9)	16.569	< 0.001
	2. Ohter common symptoms of suspected TB cases	1019(63.2)	739(61.9)	0.481	0.488
	3. Mehtods of detecting suspected TB cases	1112(68.9)	872(73.0)	5.544	0.019
	4. Screening scope of key populations for TB	230(14.3)	250(20.9)	21.590	< 0.001
	5. Screening scope of suspected DR-TB cases	824(51.1)	625(52.3)	0.436	0.509
	6. Referral of suspected TB cases	165(10.2)	159(13.3)	6.405	0.011
	7. Medical insurance reimbursement policy for suspected TB cases	262(16.2)	278(23.3)	21.887	< 0.001
	8. Scope of close contacts of TB cases	735(45.6)	522(43.7)	0.948	0.330
	Theme(A) knowledge correct rate	40.9	42.3		
B. TB treatment	9. Eligibility for free TB treatment	223(13.8)	183(15.3)	1.250	0.264
	10. Reimbursement policy for DS-TB	214(13.3)	165(13.8)	0.179	0.672
	11. Reimbursement policy for DR-TB	479(29.7)	466(39.0)	26.757	< 0.001
	12. Gold standard for TB diagnosis	897(55.6)	705(59.0)	3.304	0.069
	13. Commonly used first-line anti-TB drugs	237(14.7)	228(19.1)	9.621	0.002
	14. Common side-effects of anti-TB drugs	324(20.1)	334(28.0)	23.778	< 0.001
	15. Symptoms of hepatotoxicity adverse reactions	406(25.2)	277(23.2)	1.448	0.229
	16. Principles of TB treatment	238(14.8)	260(21.8)	23.172	< 0.001
	Theme(B) knowledge correct rate	20.0	27.4		
C. Case management	17. Eligible patients of case management	671(41.6)	364(30.5)	91.863	< 0.001
	18. Case management methods for newly diagnosed and smear-positive cases	1108(68.7)	658(55.1)	54.263	< 0.001
	19. Case management methods for newly diagnosed and smear-negative cases	302(18.7)	246(20.6)	1.544	0.214
	20. Main supervisors of case management	873(54.1)	631(52.8)	0.449	0.503
	21. Supervision contents	939(58.2)	754(63.1)	6.980	0.0088
	22. Time for sputum re-examination of newly treated cases	272(16.9)	381(31.9)	87.016	< 0.001
	23. Time for sputum examination and re-examination of re-treated cases	156(9.7)	148(12.4)	5.271	0.022
	24. Criteria for TB student patients to suspend school	1012(62.7)	651(54.4)	19.189	< 0.001
	Theme(C) knowledge correct rate	41.3	40.1		
D. TB health education	25. Disinfection manners of *Mycobacterium* TB	81(5.0)	76(6.4)	0.119	0.764
	26. Transmission route of TB	1310(81.2)	856(71.7)	35.314	< 0.001
	27. Manners of preventing the spread of TB	1511(93.7)	1055(88.4)	24.722	< 0.001
	28.TB curability	406(25.2)	425(35.6)	35.775	< 0.001
	29. Criteria for judging the infectivity of TB	824(51.1)	522(43.7)	14.917	< 0.001
	30. Identifying side-effects of anti-TB drugs	705(43.7)	437(36.6)	14.364	< 0.001
	Theme(D) knowledge correct rate	50.0	47.1		
Overall knowledge correct rate		37.3	38.7		

*Note* *TB* tuberculosis, *DR-TB *drug-resistant tuberculosis, *DS-TB *drug-susceptible tuberculosis. Percentages may not total 100 owing to rounding. Analysis through Pearson Chi-square (*χ*²) or Fisher test

Rural TB-HCWs outperformed urban peers in Case Management (41.3% vs. 40.1%; *P* < 0.05) and Health Education (50.0% vs. 47.1%; *P* < 0.001), particularly in knowledge of treatment eligibility, case management methods, and TB prevention. Conversely, urban TB-HCWs scored higher in Case Detection and Referral (42.3% vs. 40.9%; *P* < 0.05), eligibility for free TB treatment (15.3% vs. 13.8%; *P* < 0.05), especially on reimbursement policies for DR-TB (39.0% vs. 29.7%; *P* < 0.001). Knowledge of TB Treatment was uniformly poor across both groups (rural 27.4%, urban 20.0%),

### TCP implementation of TB-HCWs

Implementation of TCP practices varied between rural and urban settings (Table 4). Urban TB-HCWs generally outperformed rural counterparts, achieving national standards in First-home Visiting (≥ 90%) and Health Education (≥ 85%), and approaching compliance in Case Detection, Referral, and Tracking (≥ 80%) and Case Management (≥ 90%). In contrast, rural TB-HCWs lagged behind, especially in Case Management.

**Table 4:** Implementation practices of TB control program by the study participants, *n*/*N* (%)

Dimensions (national standard)	Service items	Primary healthcare sector levels		χ² value	*P *value
		Rural	Urban		
A. Case detection, referral and tracking (≥ 80%)	1. Screening among key populations	1200/1613(74.4)	960/1194(80.4)	13.956	< 0.001
	2. Screen for symptomatic outpatients	1346/1613(83.4)	1085/1194(90.9)	32.598	< 0.001
	3. Screen for close contacts of smear-positive patients	1184/1613(73.4)	942/1194(78.9)	11.258	0.001
	4. Referral of close contacts with suspected symptoms	1267/1613(78.5)	1026/1194(85.9)	24.984	< 0.001
	5. Referral of key population with suspected symptoms	1274/1613(79.0)	1014/1194(84.9)	16.071	< 0.001
	6. Referral of symptomatic suspected cases	1375/1613(85.2)	1105/1194(92.5)	35.536	< 0.001
	7. Ensure referrals are confirmed within 3 days	1035/1449(71.4)	689/911(75.6)	5.018	0.025
B. First-home visiting (≥ 90%)	8. Home-visit within 72 hours of notification	1407/1613(87.2)	1096/1194(91.8)	14.796	< 0.001
	9. Assign HCWs as treatment supervisors	1386/1613(85.9)	1085/1194(90.9)	15.917	< 0.001
	10. Educate patients and families on TB prevention	1409/1613(87.4)	1086/1194(91.0)	9.010	0.003
	11. Assess patient living conditions	1266/1449(87.4)	823/911(90.3)	4.853	< 0.028
C. Case management (follow-up supervision, case intervention and case closing) (≥ 90%)	12. Provide DOT during intensive phase	1113/1613(68.9)	903/1194(75.6)	15.153	< 0.001
	13. Provide DOT during continuation phase	1108/1613(68.7)	893/1194(74.8)	12.468	< 0.001
	14. Timely identification of side-effects/severe adverse reactions.	1365/1613(84.6)	1068/1194(89.4)	13.816	< 0.001
	15. Close monitoring and continued supervision for mild side-effects	1366/1613(84.7)	1073/1194(89.9)	16.156	< 0.001
	16. Intensified health education for poorly adherent patients	1233/1449(85.1)	823/911(90.3)	13.723	< 0.001
	17. Manage severe adverse reactions	1348/1613(83.6)	1069/1194(89.5)	20.372	< 0.001
	18. Report treatment interruptions exceeding one week to superiors	1236/1449(85.3)	820/911(90.0)	11.061	0.001
	19. Remind and ensure patients attend scheduled TB-designated hospital follow-up reviews	1378/1613(85.4)	1093/1194(91.5)	24.309	< 0.001
	20. Record medication adherence in treatment cards	1313/1613(81.4)	1041/1194(87.2)	16.965	< 0.001
	21. Refer patients to designated hospitals for case evaluation	1221/1449(84.3)	821/911(90.1)	16.450	< 0.001
D. Heath education (≥ 85%)	22. Provide TB health knowledge promotion among community population	1193/1449(82.3)	787/911(86.4)	6.811	0.009
	23. Health education for symptomatic outpatients	1223/1449(84.4)	818/911(89.8)	13.893	< 0.001

Note: *TB* tuberculosis, *DOT *directly observed therapy. Percentages may not total 100 owing to rounding. Analysis through Pearson Chi-square (*χ*²) or Fisher test. Percentages may not total 100 owing to rounding

Both groups performed relatively well in screening symptomatic outpatients (rural 83.4%, urban 90.9%), referring suspected cases (rural 85.2%, urban 92.5%), and providing health education for symptomatic outpatients (rural 83.4%, urban 90.9%). However, workforce-intensive services showed suboptimal implemented in both settings, notably for DOT during the intensive phase (rural 68.9%, urban 75.6%) and continuation phase (rural 68.7%, urban 74.8%). Other low-performing services included screening of close contacts of smear-positive patients (rural 73.4%, urban78.9%) and ensuring referral confirmation within 3 days (rural 71.4%, urban 75.6%).

### Factors associated with TCP implementation practices

**Fig. 3:**
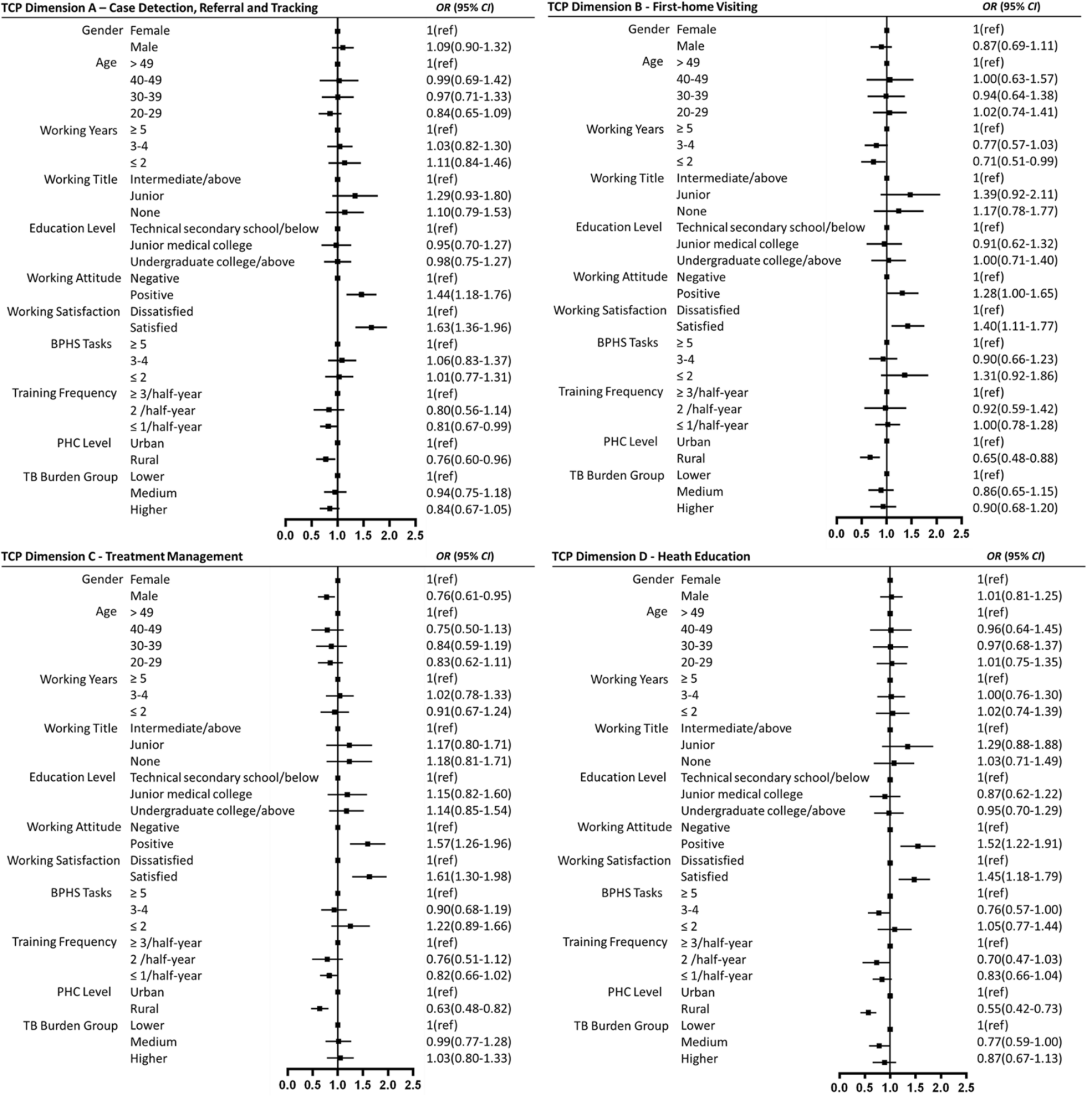
Forest plots of the multivariate logistic regression analysis for TCP implementation practices across four dimensions. **A **Case detection, referral and tracking; **B** first-home visiting; **C **treatment management; and **D **Health Education. Odds ratios (*OR*s) with 95% confidence intervals (*CI*) are shown. *TCP* tuberculosis control program, *BPHS* basic public health service, *PHC* primary healthcare, *TB* tuberculosis, *ref* reference

Multivariable analysis across four TCP dimensions identified several factors influencing implementation (Fig. 3). Positive working attitudes [odds ratio (*OR*): 1.28–1.57] and working satisfaction (*OR*: 1.40–1.63) were consistently associated with higher implementation rates. In contrast, rural setting was negatively associated with implementation across all dimensions (*OR*: 0.55–0.76, *OR* < 1 indicates lower likelihood of achieving high TCP implementation). Other negative predictors included: infrequent training (≤ 1 per half-year) for Case Detection, Referral and Tracking [*OR* = 0.81, 95% *CI*: 0.67–0.99]; limited PHC experience (≤ 2 year) for First-home Visits [*OR* = 0.71, 95% *CI*: 0.51–0.99]; male gender for Case Management [*OR* = 0.76, 95% *CI*: 0.61–0.95]; moderate BPHS task load (3–4 tasks) [*OR* = 0.76, 95% *CI*: 0.57–1.00)]; and working in medium incidence areas [*OR* = 0.77, 95% *CI*: 0.59–1.00] for Health Education implementation.

### Perceived concerns to TCP implementation

**Fig. 4:**
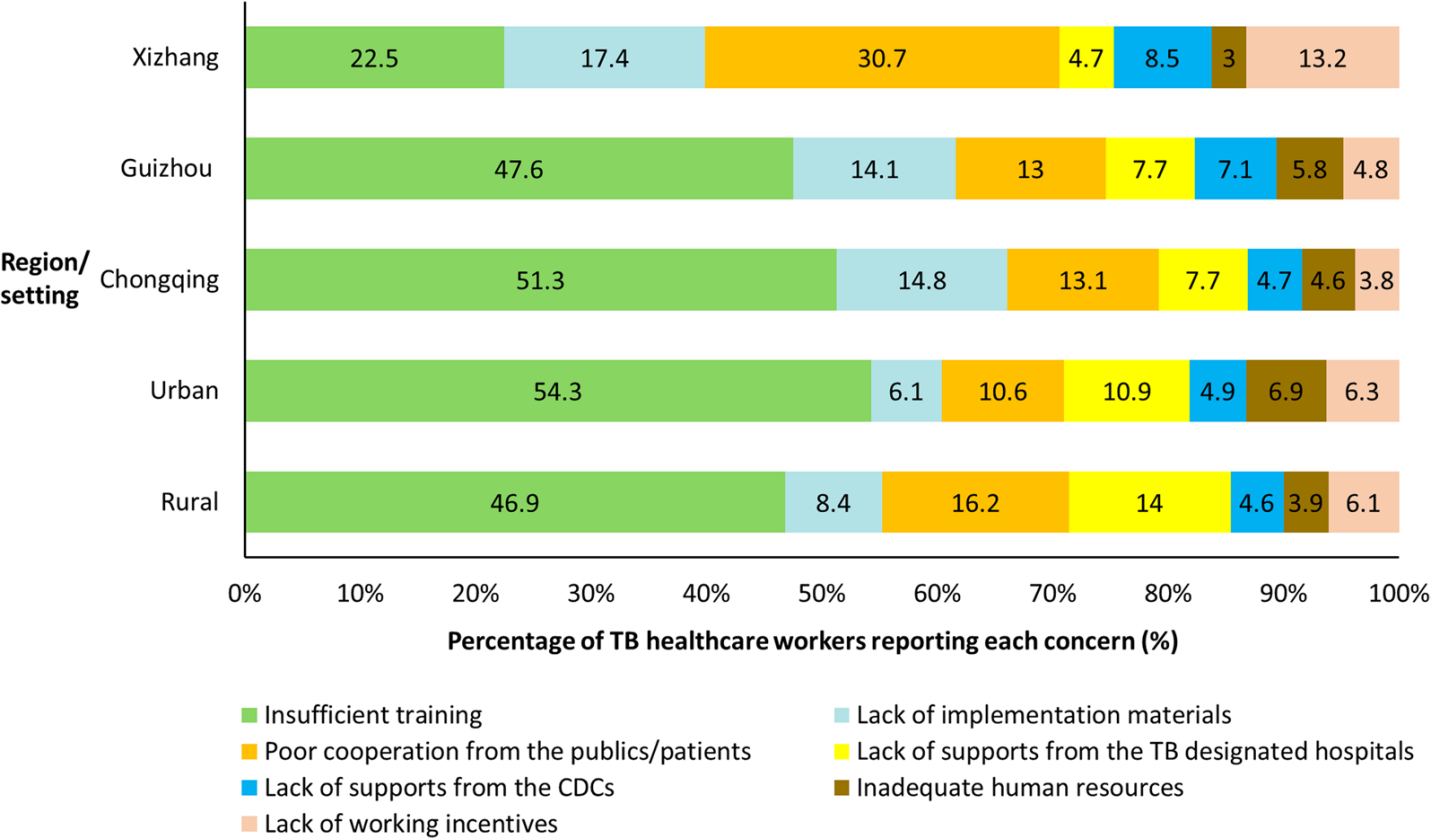
Perceived concerns regarding TB control program implementation among TB healthcare workers (*n *= 2807). Stacked bar chart showing the proportion (%) of different concerns perceived by TB healthcare workers, stratified by region (Xizang, Guizhou, and Chongqing) and by setting (rural and urban). *CDC* Centers of Disease Control, *TB* Tuberculosis

Figure 4 summarizes the concerns perceived by TB-HCWs’ regarding TCP implementation. The most common issues were insufficient training, poor patient or public cooperation, and limited support from TB-designated hospitals. Urban TB-HCWs more frequently reported training deficiencies (51.3% vs. 46.9% rural), while rural TB-HCWs emphasized non-cooperation from patient and public (16.2% vs. 10.6% urban), and insufficient institutional support from TB-designated hospital (14.0% vs. 10.9% urban). Regionally, training inadequacies were most often reported in Chongqing (51.3%) and Guizhou (47.6%), but less so in Xizang (22.5%). Conversely, TB-HCWs in Xizang more frequently reported patient and public non-cooperation (30.7%), and lack of working incentives (13.2%), concerns less prominent in the other two regions.

## Discussion

Effective TB control in PHC settings relies on the integration of three core elements—case detection and referral, patient-centered case management, and health education—which form the backbone of the WHO’s End TB Strategy. For TB-HCWs in PHC sectors, having adequate knowledge of TB care is foundational for delivering high-quality, patient-centered care, and translating knowledge into practice. In this study, we developed a 30-item TB knowledge assessment questionnaire for TB-HCWs and comprehensively evaluated their knowledge, practices, and perceived concerns regarding TCP implementation across three PLADs in western China.

Consistent with previous studies, our findings revealed low overall TB knowledge among TB-HCWs (38.4%) [9, 33–38]. Several treatment- and screening-related items showed particularly poor correct rate (< 20%). These deficiencies are concerning since they have direct implications for treatment outcomes and public health. Limited knowledge of disinfection methods increases transmission risk; inadequate awareness of free treatment eligibility and reimbursement policies may delay care-seeking; and insufficient knowledge of first-line anti-TB drugs and treatment principles undermines adherence and reduces treatment success. Addressing these critical gaps is essential for effective TCP implementation and for strengthening patients’ confidence in PHC-based TB services.

Rural-urban differences were evident. Rural TB-HCWs demonstrated better knowledge in Case Management and Health Education, whereas urban TB-HCWs scored higher in Case Detection and TB Treatment. These differences might reflect variations in working roles and service contexts. Rural TB-HCWs, deeply embedded in communities, undertook multiple BPHS tasks and broader TB care responsibilities, including follow-up, health promotion, and household-level services. This close patient contact may partly compensate for limited formal training. In contrast, urban TB-HCWs generally possessed higher educational attainment and greater access to professional training, enhancing their knowledge in clinically oriented domains such as TB treatment. Urban TB-HCWs may also face additional challenges from large migrant populations and higher volumes of suspected TB cases, necessitating stronger diagnostic and case detection competencies.

Overall, TB-HCWs’ knowledge and training in TCP remained inadequate, highlighting the need for more practical, tailored, and accessible training programs—particularly in resource-limited rural settings. Previous studies underscored the value of well-designed TB training while identifying common shortcomings in content, duration, and delivery that contributed to inadequate competency and job dissatisfaction [9, 18, 30, 39]. Our findings suggested that TCP training should be refined, sustained and people-centered. Content and frequency should be dynamically adapted to regional and workforce needs. Digital, modular platforms could expand access and flexibility, especially in remote areas. In addition, incorporating communication and counselling skills to strengthen patient-centered care would further enhance TCP effectiveness.

Consistent with previous studies, our findings revealed marked rural-urban differences in TCP implementation [18, 30, 33, 39]. Although overall performance fell short of Chinese national standards, urban TB-HCWs generally achieved higher compliance, meeting targets in First-home Visiting (≥ 90%) and Health Education (≥ 85%), and approaching benchmarks in Case Detection, Referral, and Tracking (≥ 80%) and Case Management (≥ 90%), reflecting stronger institutional support [3]. In contrast, workforce-intensive services, particularly DOT, were implemented suboptimally in both settings. As reported previously, Case Management, the most labor-intensive TCP component, remained challenging due to limited patient-centered approaches, poor patient cooperation, and low health literacy, insufficient workforce, and persistent social stigma [9, 18, 30, 33, 39]. Traditional DOT may not be suitable for all patients and conflicts with principles of patient-centered care [56–59]. Although digital adherence technologies (e.g., smart pillboxes, video-observed therapy, short message service reminders) have been explored, systematic reviews indicated inconsistent effectiveness [60]. These findings underscored the need for flexible, context-specific strategies to promote patient-centered supports, particularly in high-burden, resource-constrained areas. Individualized adherence management approaches, such as combining face-to-face follow-up, telephone calls, and text messaging for elderly patients, and digital health approaches for younger populations, could improve adherence while reducing TB-HCW workload. Furthermore, fostering patient engagement as active partners in care and empowering TB-HCWs to reflect on challenges and contribute to service design are vital for sustainable TCP improvement.

Our analysis further revealed that positive working attitudes and working satisfaction were associated with better TCP implementation, whereas rural setting, infrequent training, and limited PHC experience emerged as barriers. Reported concerns—inadequate training, poor patient cooperation, and weak coordination with TB-designated hospitals—mirrored structural challenges documented in China and other high-burden countries [9, 18, 30, 39]. These findings highlighted several priorities for strengthening PHC-based TCP. First, training should be more practical, frequent, and context-specific, particularly in rural areas, with emphasis on critical knowledge gaps such as TB treatment, infection control, and reimbursement policies. Modular digital platforms could improve access in remote areas, while integrating patient-centered communication skills would enhance HCW-patient interaction and cooperation. Second, workforce and system-level supports are essential, including sustainable incentive mechanisms, clear career development pathways, and stronger collaboration between PHC sectors and TB-designated hospitals. Third, differentiated patient management approaches—ranging from face-to-face or phone-based follow-up for rural elderly patients to app-based adherence tools for younger urban patients—may better align with patient needs and address workload burden. Finally, community-based TB health promotion and stigma reduction initiatives are essential to enhance patient cooperation and treatment adherence.

This study developed a structured TB knowledge assessment questionnaire that could be adapted for evaluating TCP competency in PHC settings. It also systematically assessed knowledge, practices, and concerns of TB-HCWs using a large sample from high-TB burden, resource-limited regions, offering a holistic view of the multifaceted challenges in PHC-based TCP implementation. Nervertheless, several limitations should be acknowledged. First, the cross-sectional design precludes causal inference. Second, voluntary participation and self-reported data may have introduced recall, selection, and social desirability biases, as TB-HCWs more motivated or engaged in TB control may have been overrepresented and prone to over-report positive behaviors. Third, the purposive selection of three provincial regions using a non-probability sampling approach limited generalizability. Fourth, although reliability was confirmed, the TB knowledge assessment questionnaire had not yet undergone full validation (e.g., face, criterion, and construct validity). Future research will address these aspects to refine the instrument into a standardized tool for broader application. Finally, this study focused solely on provider perspectives without incorporating patient viewpoints. Further studies should therefore engage multiple stakeholders to generate more comprehensive evidence to inform effective and equitable TB control strategies.

## Conclusions

This study identified substantial gaps in TB knowledge and practice among PHC-based TB-HCWs in western China, alongside clear rural-urban disparities and obstacles. Tailored interventions are urgently needed. Training programs should be context-specific, frequent, and patient-centered; flexible, digitally supported adherence management strategies should be adopted to address diverse patient needs; and stronger work incentives and multisector coordination are essential to promote implementation efficiency. Future longitudinal research is warranted to evaluate the effectiveness and sustainability of these interventions and to accelerate progress toward national and global End TB targets.

## Supplementary Information

Additional file 1.

## Data Availability

The datasets and the questionnaires used and/or analyzed during the current study are available from the corresponding author upon reasonable request.

## Abbreviations

TB: Tuberculosis
MDR/RR-TB: Multidrug-resistant or rifampicin-resistant TB
DS-TB: Drug-susceptible tuberculosis
WHO: World Health Organization
DOT: Directly observed therapy
CDC: Center for Disease Control
PHC: Primary healthcare
TCP: Tuberculosis control program
HCW: Healthcare worker
PHC-based TCP: Tuberculosis control program services delivered by healthcare workers in PHC sectors
DR-TB: Drug-resistant tuberculosis
BPHS: Basic public health service
PLAD: Provincial-level administrative divisions
STROBE: Strengthening the Reporting of Observational Studies in Epidemiology
pre-XDR-TB: Pre-extensively drug-resistant TB
*OR*: Odds ratio
